# Directing Stem Cell Commitment by Amorphous Calcium Phosphate Nanoparticles Incorporated in PLGA: Relevance of the Free Calcium Ion Concentration

**DOI:** 10.3390/ijms21072627

**Published:** 2020-04-09

**Authors:** Olivier Gröninger, Samuel Hess, Dirk Mohn, Elia Schneider, Wendelin Stark, Sonja Märsmann, Petra Wolint, Maurizio Calcagni, Paolo Cinelli, Johanna Buschmann

**Affiliations:** 1 Institute for Chemical and Bioengineering, Department of Chemistry and Applied Biosciences, ETH Zurich, 8093 Zurich, Switzerland; olivier.groeninger@chem.ethz.ch (O.G.); samuel.hess@chem.ethz.ch (S.H.); dirk.mohn@chem.ethz.ch (D.M.); elia.schneider@chem.ethz.ch (E.S.); wendelin.stark@chem.ethz.ch (W.S.); 2 Department of Conservative and Preventive Dentistry, Center of Dental Medicine, University of Zurich, 8032 Zurich, Switzerland; 3 Division of Plastic Surgery and Hand Surgery, University Hospital Zurich, 8091 Zurich, Switzerland; sonja.maersmann@usz.ch (S.M.); Petra.Wolint@usz.ch (P.W.); maurizio.calcagni@usz.ch (M.C.); 4 Division of Trauma Surgery, University Hospital Zurich, 8091 Zurich, Switzerland; paolo.cinelli@usz.ch

**Keywords:** adipose-derived stem cells, PLGA, amorphous calcium phosphate nanoparticles, calcium ions, gene expression

## Abstract

The microenvironment of mesenchymal stem cells (MSCs) is responsible for the modulation in MSC commitment. Nanocomposites with an inorganic and an organic component have been investigated, and osteogenesis of MSCs has been attributed to inorganic phases such as calcium phosphate under several conditions. Here, electrospun meshes and two-dimensional films of poly(lactic-co-glycolic acid) (PLGA) or nanocomposites of PLGA and amorphous calcium phosphate nanoparticles (PLGA/aCaP) seeded with human adipose-derived stem cells (ASCs) were analyzed for the expression of selected marker genes. In a two-week in vitro experiment, osteogenic commitment was not found to be favored on PLGA/aCaP compared to pure PLGA. Analysis of the medium revealed a significant reduction of the Ca^2+^ concentration when incubated with PLGA/aCaP, caused by chemical precipitation of hydroxyapatite (HAp) on aCaP seeds of PLGA/aCaP. Upon offering a constant Ca^2+^ concentration, however, the previously observed anti-osteogenic effect was reversed: *alkaline phosphatase*, an early osteogenic marker gene, was upregulated on PLGA/aCaP compared to pristine PLGA. Hence, in addition to the cell–material interaction, the material–medium interaction was also important for the stem cell commitment here, affecting the cell–medium interaction. Complex in vitro models should therefore consider all factors, as coupled impacts might emerge.

## 1. Introduction

Discovering microenvironments that direct the osteogenic commitment of stem cells is currently one of the central topics in the field of bone repair and regeneration. Supplements, such as calcium ions [1], bone morphogenetic proteins (BMPs) [2], dexamethasone [3] and bioactive peptides [4,5] in the culture media of stem cells trigger the intended differentiation towards the osteoblast phenotype [6]. Apart from adding supplements, co-cultures [7], microtissues [8] or mechanical stimulation [9,10,11] are promising approaches in this regard. Moreover, the composition of a material acting as a scaffold [12], its surface properties [13], pore size and pore connectivity [14] are essential for the specific commitment of stem cells.

With the aim of mimicking natural bone, its composition and structure has been profoundly analyzed [15,16]. From the knowledge obtained, fabrication of biomaterials resembling bone structure could be further developed, not only for tissue engineering but also for coating of inert implant materials [17]. A mineralized collagen fibril forms the main building block of bone tissue, with the most abundant mineral phase consisting of hydroxyapatite (HAp) [18]. On the other hand, the organic phase is primarily composed of collagen I, which constitutes approximately 20 wt% of the bone substance [19]. Hence, implant materials intended for bone regeneration often include a mineral and an organic component, among others [20]. The use of calcium phosphate compounds as inherent entities in bone void fillers or scaffold materials aimed at tissue engineering has recently gained considerable interest. Several reports on novel materials containing calcium phosphate nanoparticles elucidate their potential [21,22,23,24,25,26,27]. The inorganic phase incorporated within an organic carrier may act beneficially in terms of osteogenic commitment; for example, fibroblast-derived extracellular matrix (ECM) sheets for the replacement of the periosteum promoted osteogenic differentiation of human mesenchymal stem cells (MSCs) [28] when covered with calcium phosphate particles. Another study reports the effect of biphasic calcium phosphate (BCP) nanoparticles embedded in polyvinyl alcohol/gelatin [29]. The presence of those nanoparticles affected the protein expression of MG-63 cells by upregulation of collagen I and osteopontin as early as 3 and 7 days in vitro. Moreover, in vivo application revealed the beneficial effect of these inorganic nanoparticles: in a rat model, 50 wt% of BCP resulted in increased bone formation 2 and 4 weeks post-implantation [29]. Nevertheless, the impact on the commitment of MSCs may vary depending on the exact chemical composition, crystallinity, solubility and size of the calcium phosphate particles included in the organic phase.

In our previous work, we focused on a nanocomposite based on poly(lactic-co-glycolic acid) (PLGA) and amorphous calcium phosphate nanoparticles (PLGA/aCaP, weight ratio 60:40) seeded with human adipose-derived stem cells (ASCs) [11,30,31,32]. Moreover, the gene expression of primary human ASCs seeded on a thin casted film made of either PLGA or PLGA/aCaP or on electrospun random fiber meshes (PLGA or PLGA/aCaP) was assessed to investigate the effects of the structure and material composition [33].

The goal of the current study was to evaluate the impact of aCaP nanoparticles in PLGA composites on the gene expression of ASCs when seeded on the materials in vitro. The exposition of calcium phosphate nanoparticles to ASCs, either in a 2D or in a 3D microenvironment, may affect their commitment.

The hypotheses were that

(i) aCaP nanoparticles in PLGA evoke the osteogenic commitment of ASCs in basal medium;
(ii) osteogenic commitment—as evoked by aCaP nanoparticles—is enhanced in osteogenic induction medium;
(iii) 3D fiber meshes of PLGA/aCaP evoke osteogenic commitment even to a greater extent than do corresponding 2D flat films of PLGA/aCaP.

## 2. Results

### 2.1. Material Characterization

**Figure 1:**
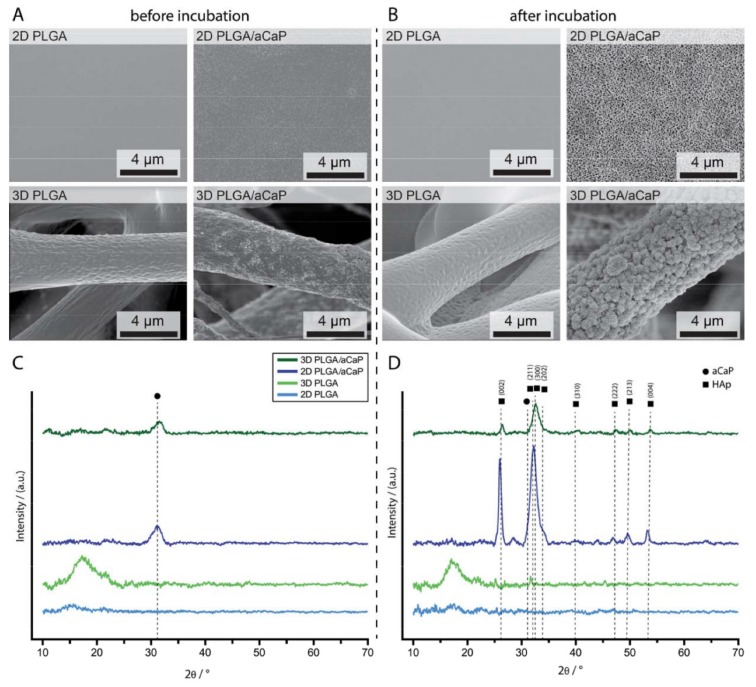
SEM images of the surface of poly(lactic-co-glycolic acid) (PLGA) and amorphous calcium phosphate nanoparticles (PLGA/aCaP) fabricated as films or electrospun meshes (**A**). On the surface, the films made of PLGA are flat, in contrast to the PLGA/aCaP films, where the nanoparticles can be seen quite well (increased magnification in Figure S1). The surface of the scaffolds was analyzed by means of SEM after (**B**) incubation for 2 weeks in DMEM at 37 °C (**B**). Hydroxyapatite crystals can be observed on 2D and 3D PLGA/aCaP scaffolds. The formation of hydroxyapatite on material with incorporated aCaP nanoparticles was confirmed by X-ray diffraction (XRD) pattern ((**C**): before incubation; (**D**): after incubation) [35]. The black dot denotes the aCaP phase.

First, the materials without cells were characterized by means of SEM and X-ray diffraction (XRD) (Figure 1 and supporting information Figure S1). The aCaP nanoparticles are visible before incubation on PLGA/aCaP scaffolds using SEM (Figure 1A) and its amorphous phase is confirmed by XRD (Figure 1C). Upon incubation in culture medium without cells, crystalline hydroxyapatite (Figure 1B,D) was formed only on composites containing aCaP nanoparticles. X-ray measurements show the shift from a wide peak assigned to amorphous aCaP (Figure 1C) [34] to the sharp crystalline peaks of HAp (Figure 1D) [35].

### 2.2. Seeding Adipose-Derived Stem Cells and Gene Expression Analysis

**Figure 2:**
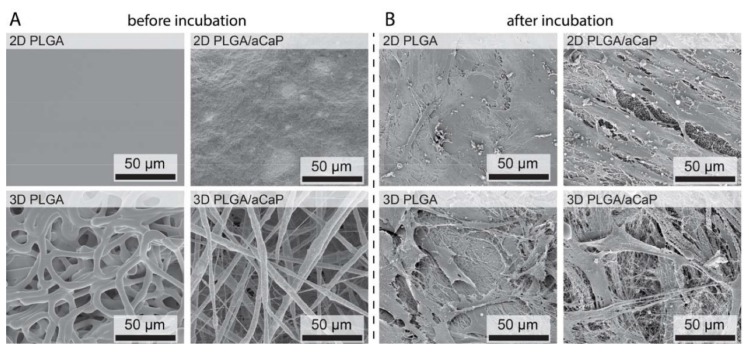
The 2D and 3D scaffolds of PLGA and PLGA/aCaP, shown without cells in (**A**), were seeded with adipose-derived stem cells for 14 days, shown in (**B**). The interaction between cells and material seems to be enhanced on scaffolds containing calcium phosphate compounds because of aCaP nanoparticles and newly formed hydroxyapatite (Hap), while biofilm formation is observed on pristine PLGA rather than adhesion of the cells as found for PLGA/aCaP.

**Figure 3:**
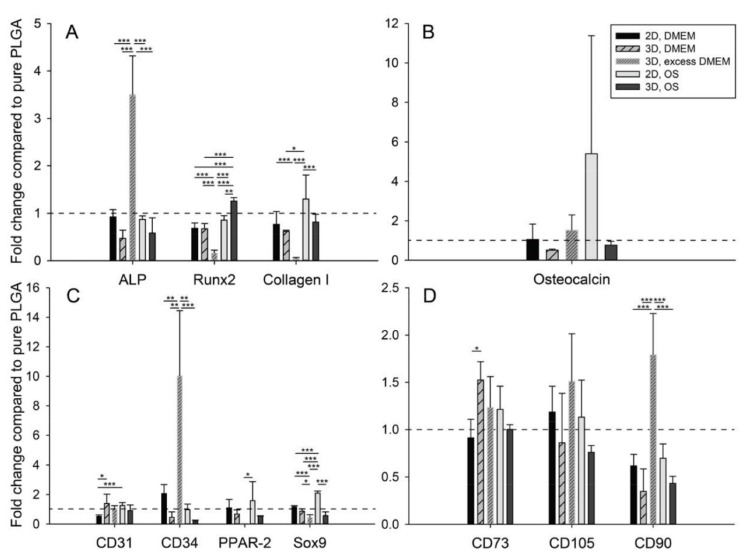
Manifold gene expression of human adipose-derived stem cells (ASCs) seeded on PLGA or on PLGA/aCaP realized as casted films or as electrospun random fiber meshes. The fold change of aCaP-seeded PLGA compared to pure PLGA is shown in (**A**) ALP, Runx2 and collagen I, (**B**) osteocalcin, (**C**) CD31, CD34, PPAR-γ-2 and Sox9, (**D**) CD 73, CD105 and CD 90. Values at 1.0 (dashed line) indicate no change in gene expression, whereas higher values indicate enhanced gene expression if aCaP nanoparticles are present. Key: OS = osteogenic medium. The *p*-values of pairwise comparison of one-way ANOVA statistics were considered significant if *p* < 0.05, denoted by *. **, *p* < 0.01; ***, *p* < 0.001. For significant differences between PLGA/aCaP and PLGA, we refer to SI Figures S1 and S2.

Then, the scaffolds were seeded with human adipose-derived stem cells (Figure 2). After two weeks, osteogenic marker genes *ALP*, *Runx2*, *collagen I* and *osteocalcin* were analyzed. Moreover, in addition to minimal stem cell markers *CD73, CD90* and *CD105*, some other genes were selected, such as *CD31* and *CD34* (endothelial), *PPAR-γ-2* (adipogenic) and *Sox9* (chondrogenic). The presence of the aCaP nanoparticles in PLGA led to a significant downregulation of osteogenic markers genes (Figure 3 and supporting information Figure S2). In addition to *ALP, Runx2* and *collagen I* expression (3D, DMEM and 3D, OS), *osteocalcin* (3D, DMEM) was also reduced. There was no significant upregulation of osteogenic marker genes—neither for the two architectures (2D and 3D) nor for the two culture media (DMEM and OS).

The analysis of the changes in gene expression for other markers revealed a significant upregulation for *CD31* expression in 3D, DMEM as well as for *CD34* expression in 2D, DMEM, but also a significant downregulation for *CD31* in 2D, DMEM and for CD34 in 3D, DMEM. In osteogenic medium, *CD31* and *CD34* genes were unaffected. There was also no change in the gene expression of *PPAR-γ-2* and *Sox9*, neither for 2D and 3D nor for the two culture media.

Stem cell markers *CD73* and *CD90* were significantly affected when seeded on the nanocomposite compared to pure PLGA. While *CD73* experienced an upregulation in 3D, DMEM and 3D, OS, *CD90* was downregulated in DMEM (both 2D and 3D) and in 3D, OS.

### 2.3. Free Calcium Ion Concentration

**Figure 4:**
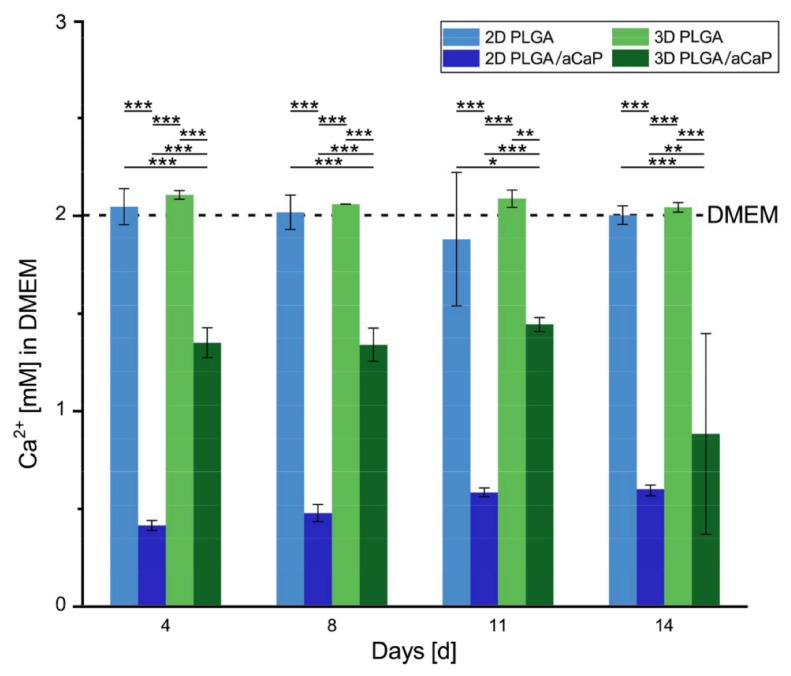
Ca^2+^ ion concentration of DMEM medium exposed to either PLGA or PLGA/aCaP random fiber meshes and films. On PLGA/aCaP materials, calcium ions are reduced in 2D film to 0.5 mM and in 3D electrospun meshes to 1.3 mM. The *p*-values of pairwise comparison of one-way ANOVA statistics were considered significant if *p* < 0.05, denoted by *. **, *p* < 0.01; ***, *p* < 0.001.

As a further step, cell-free 2D films and 3D electrospun meshes of PLGA ± aCaP nanoparticles were incubated in DMEM to gain further insights into the interaction between the medium and material itself (no cells present). The concentration of free calcium ions in the supernatant was measured before and after every medium change (Figure 4). The Ca^2+^ concentration in the supernatant of calcium phosphate-containing composites was reduced significantly after incubation at 37 °C for up to 14 days (medium change time interval: 3 or 4 days), while pristine PLGA did not indicate any change compared to the basal concentration in the medium.

**Figure 5:**
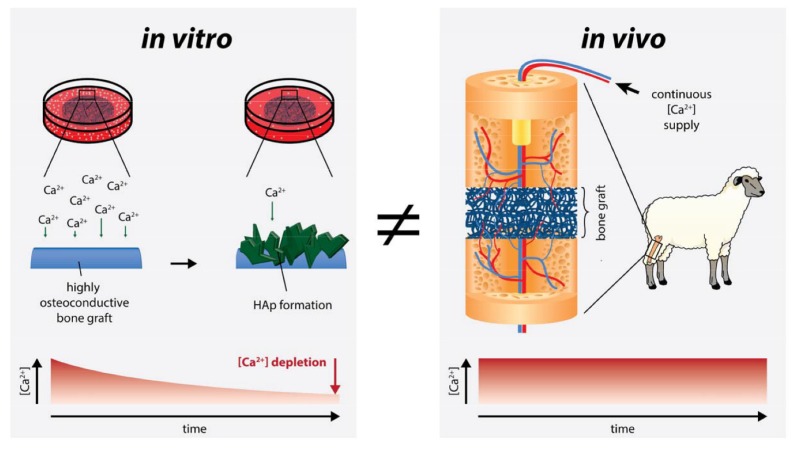
Studying potential bone grafts is often performed under in vitro conditions. Highly osteoconductive materials lead to the rapid formation of calcium phosphate crystal hydroxyapatite (HAp). In consequence, the free calcium ion in the medium depletes. In such cases, in vitro studies are not able to mimic in vivo conditions anymore, since continuous supply of minerals, such as Ca^2+^, is given by diffusion under in vivo conditions.

In vivo, calcium ions are continuously delivered by diffusion and a constant level remains over time (Figure 5). In our specific system, however, the formation of HAp and other calcium phosphate phases led to a decrease in free calcium ion concentration (Figure 4 and Figure 5).

**Figure 6:**
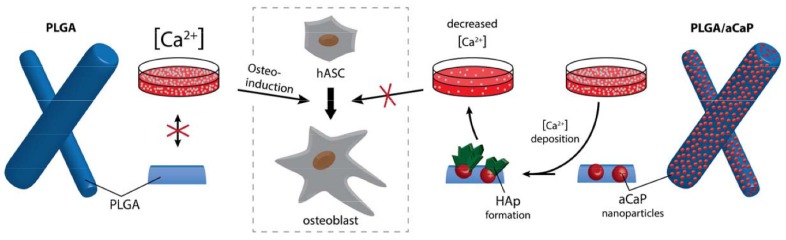
The interaction of material and medium is illustrated and influences several cellular pathways leading to distinct cell differentiation. Bone scaffolds with incorporated calcium phosphate nanoparticles induce the formation of hydroxyapatite, and thus the deposition of calcium ions decreases free calcium in the medium, which reduces osteoinduction significantly. On pure PLGA, no interaction of material and medium, such as mineral precipitation, is observed and therefore leads to usual calcium ion concentrations and normal osteoinduction.

Calcium ion levels in the medium in contact with only PLGA was measured to be approximately 2.0 mM, which is the usual Ca^2+^ concentration in DMEM with 10% fetal bovine serum (FBS) (basal medium) after 3 days of incubation time (Figure 4). ACaP nanoparticles can act as initial nuclei for crystallization and precipitation of inorganic phases composed of calcium and phosphate (Figure 6). Consequently, the Ca^2+^ in the supernatant of the calcium phosphate-containing material was reduced to 0.52 ± 0.08 mM (2D) resp. 1.33 ± 0.17 mM (3D), a 3.8-fold (2D) and 1.5-fold (3D) reduction, respectively. Normalized to the weight of the discs, the decrease is 0.08 mM (2D) and 0.24 mM (3D) per µg of incubated material. Hence, the 3D electrospun meshes depleted their supernatant medium 3-fold more efficiently than the 2D films.

### 2.4. Providing Excess Culture Medium 

In light of these findings, further experiments with 3D electrospun meshes in excess medium were conducted, i.e., 10-fold more medium, warranting a constant free calcium ion concentration (supporting information Figure S3). Under these excess medium conditions, gene expression revealed that ALP was significantly upregulated in PLGA/aCaP compared to PLGA (Figure 3). This can be confirmed by the significant lower ΔCT of the PLGA/aCaP 3D, excess DMEM compared to PLGA 3D, DMEM (supporting information Figure S4).

While *CD31* expression was significantly downregulated on films in DMEM and upregulated in 3D meshes in DMEM (non-excess), it was not affected in the excess system. *CD34* expression, however, was significantly upregulated by a factor of 10 in the presence of aCaP when the cell-seeded meshes were cultivated in excess DMEM (Figure 3). The analysis of *PPAR-γ-2* and *Sox9* as representative genes for adipogenic and chondrogenic commitment, respectively, revealed a significant downregulation when seeded on PLGA/aCaP compared to PLGA (Figure 3). Finally, under excess medium conditions and with regard to stem cell markers, only *CD73* and *CD90* were significantly upregulated; *CD105* was not affected (Figure 3).

### 2.5. Mineralization

**Figure 7:**
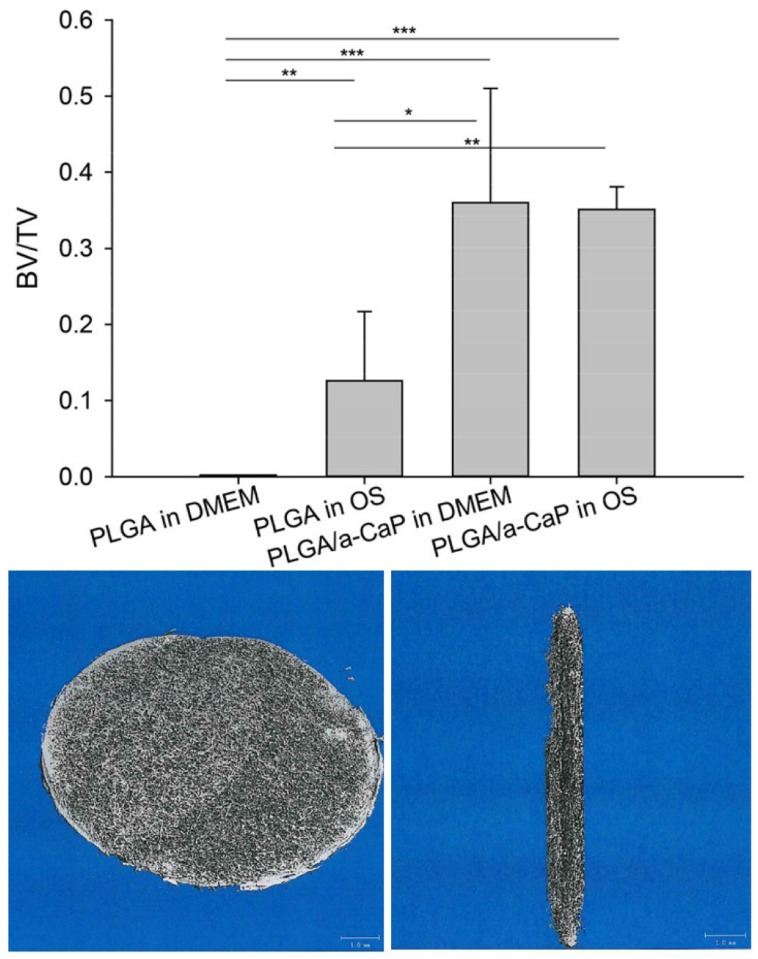
The bone volume (BV)/total volume (TV) (= bone volume fraction) is given for the two different materials (PLGA and PLGA/aCaP) in the two different culture media (DMEM and osteogenic medium = OS). The bone volume fraction (BV/TV) was assessed for the ASC-seeded three-dimensional random fiber meshes after 14 days cultured in vitro (see Materials and Methods). Below, there are typical micro-CT images of a PLGA scaffold: surface (left) and cross-section (right), scale bars = 1 mm. The *p*-values of pairwise comparison of one-way ANOVA statistics were considered significant if *p* < 0.05, denoted by *. **, *p* < 0.01; ***, *p* < 0.001.

Micro-CT analysis of 3D random fiber meshes revealed that the bone volume (BV) fraction increased in OS compared to DMEM when PLGA was the material of choice; however, there was no increase in the BV fraction between OS and DMEM when PLGA/aCaP was used (Figure 7).

## 3. Discussion

The microenvironment of stem cells directs their fate with regard to differentiation [36]. In order to elucidate the effect of aCaP nanoparticles incorporated in PLGA on the gene expression of human stem cells, 3D electrospun meshes and 2D films made of PLGA and PLGA/aCaP, respectively, were used and cultivated either in basal or in osteogenic culture medium. After two weeks, a time point when the changes in commitment were hypothesized to become evident, osteogenic marker genes *ALP*, *Runx2*, *collagen I* and *osteocalcin* were analyzed. During the time course of osteogenic differentiation, early upregulated genes include *ALP* [37], *Runx2*, followed by *collagen I* and later *osteocalcin* expression. In contrast to our expectations, the presence of the aCaP nanoparticles in PLGA led to a significant downregulation of these osteogenic markers.

Although previous in vivo studies in rabbits [38] and sheep [39] clearly demonstrated that the PLGA/aCaP nanocomposite acted as osteoconductive materials and supported bone regeneration as a bone void filler, the exposure of human ASCs seeded on PLGA/aCaP in vitro showed the opposite—based on gene expression results, there was an anti-osteogenic commitment.

As calcium plays a pivotal role in several signaling pathways [40,41], changing free calcium ion levels may lead to other results that do not represent in vivo conditions. Enhanced Ca^2+^ deposition, which is the case for highly osteoconductive materials [42], may lead to Ca^2+^ depletion in vitro if there is not sufficient Ca^2+^ available to replenish the deficit; however, in vivo, the calcium concentration is generally maintained at constant as Ca^2+^ is continuously delivered to sites with transiently lowered concentrations [43].

Therefore, cell-free 2D films and 3D electrospun meshes of PLGA ± aCaP nanoparticles were incubated in DMEM and analysis of the supernatant revealed a significant depletion of the free Ca ion concentration in case of the nanocomposite PLGA/aCaP; however, this was not the case for pure PLGA, where no changes in free Ca ion concentration were found. Obviously, the in vitro conditions used in this study differed from typical in vivo conditions with respect to free calcium ion levels; in vivo, calcium ions are continuously delivered by diffusion, leading to a constant level over time. In our specific system, however, the formation of HAp and other calcium phosphate phases led to a decrease in free calcium ion concentration, which affected the commitment of the ASCs.

In light of these findings, further experiments with 3D electrospun meshes in excess medium were conducted, i.e., 10-fold more medium, warranting a constant free calcium ion concentration. As culture medium, DMEM was chosen because it was hypothesized that it was more difficult to reverse the observed anti-osteogenic effect by providing a constant Ca^2+^ concentration in basal medium DMEM than in osteogenic induction medium. The 3D meshes were used as they had been shown to induce a 3-fold higher free calcium depletion per mass of scaffold compared to the 2D films.

As originally expected, gene expression revealed that *ALP* was significantly upregulated in PLGA/aCaP compared to PLGA after two weeks. *ALP* is an early osteogenic marker, usually upregulated within the first week after incubation with OS. However, as we used basal medium without further supplementation, it seemed reasonable that it was upregulated after 2 weeks. Thus, an early osteogenic commitment was confirmed—evoked by the presence of aCaP nanoparticles in PLGA. The other osteogenic markers analyzed here, Runx2 and collagen I, still experienced a downregulation, as was the case under non-excess conditions, while osteocalcin was not affected. While upregulation of Runx2 had been expected because it is known to be responsible for (i) the pre-osteoblastic commitment of stem cells [44] and (ii) the activation of *ALP* during pre-osteoblastic differentiation [45], the osteogenic commitment of collagen I and osteocalcin usually appears later during the osteogenic differentiation process, explaining these findings. For *ALP*, however, the expected upregulation in the presence of aCaP nanoparticles incorporated in PLGA was confirmed.

In addition, one remarkable change in gene expression under excess medium conditions was the 10-fold upregulation of *CD34*. This significant *CD34* upregulation on meshes in excess DMEM in the presence of aCaP nanoparticles may have induced other functional cues in addition to the commitment towards an endothelial cell type; induction of *CD34* gene expression in ASCs might also be attributed to replicative capacity and stemness in addition to endothelial cell-related pathways [46]. In addition, although most of the ASC subpopulations are CD34^-^, it has been reported that approximately 8% of the cells may be CD34^+^. As shown for another source of mesenchymal stem cells from peripheral blood, CD34^+^ stem cells are easily differentiated towards the osteoblastic phenotype. It is noteworthy to mention that in excess DMEM, *CD31* did not experience an upregulation, while there was this high impact on *CD34*. Aguirre et al. have shown that CD34^+^ progenitor cells from bone marrow had a weak *CD31* expression, but nevertheless could differentiate into mature endothelial cells [47].

The analysis of *PPAR-γ-2* and *Sox9* as representative genes for adipogenic and chondrogenic commitment, respectively, revealed a significant downregulation when seeded on PLGA/aCaP compared to PLGA under excess medium conditions. These findings are coherent with the initial commitment of the ASCs found for the osteogenic phenotype; while *ALP* was significantly upregulated, adipogenic and chondrogenic commitment were suppressed.

As for the impact of aCaP nanoparticles in PLGA meshes and in the presence of a constant calcium ion concentration, only *CD73* and *CD90* were significantly upregulated; *CD105* was not affected. Although *CD90* was downregulated in the systems investigated under calcium depletion, it was upregulated in excess DMEM. During differentiation, typical stem cell markers should theoretically be downregulated, as the gene expression changes and stemness is lost upon lineage commitment. Interestingly, we found an upregulation here, which might show that these stem cell marker genes are sensitive towards calcium in a different way.

Finally, the micro-CT analysis revealed that the BV fraction increased in OS compared to DMEM when PLGA was used, but not between DMEM and OS for PLGA/aCaP. Hence, the difference in BV/TV between PLGA/aCaP and PLGA was larger in DMEM than in OS. Combining results from cellular and non-cellular incubation leads to the conclusion that calcium phosphate phases produced by cells are only reflected in BV fractions on PLGA (where BV/total volume (TV) OS > DMEM). In contrast, on PLGA/aCaP, the contribution of cells to BV/TV is superimposed by (i) aCaP nanoparticles already incorporated and (ii) chemical HAp precipitation of calcium ions from the medium induced by the nanoparticles, levelling off BV/TV to approximately 0.35. Moreover, dissolution of calcium phosphate phases should not be neglected and may add to these findings of similar BV/TV for PLGA/aCaP in both culture media.

Many studies hypothesized that the presence of 40 wt% aCaP nanoparticles incorporated in PLGA either realized as casted films (2D) or as electrospun random fiber meshes (3D) would promote the osteogenic commitment of ASCs, as observed in previous preclinical studies in vivo. In contrast, when using a classical in vitro setup with limited fluidic exchange, we found an overall downregulation of early osteogenic markers, with some exceptions—and these exceptions were always found for the 2D system realized by casted films. We analyzed the supernatant medium for calcium levels as the direct local interaction of cells with the substrate is not the only important factor for stem cell fate. It was found that the presence of aCaP nanoparticles significantly decreased the free calcium ion concentration. Hence, extracellular calcium levels and, consequently, intracellular calcium levels might have a more important impact on the modulation of stem cell fate as compared to direct interactions with inorganic nanoparticles incorporated in PLGA (solid phase). Medium composition and substrate composition are coupled variables because they influence each other and, together, they affect the gene expression of the cells in contact with both the substrate and the medium, respectively. We were able to compensate and attenuate the decrease in free calcium ion concentration by a 10-fold increase in the medium volume. Under this high ratio of culture medium-to-scaffold volume, we found the typical increase in *ALP* gene expression in the presence of aCaP nanoparticles. Together, starting osteogenesis and the commitment of a subpopulation towards the endothelial cell type—which had been shown for the chosen cell line before [31]—established PLGA/aCaP as a good bone biomimetic scaffold under in vitro conditions without further medium supplementation, providing constant extracellular calcium concentrations.

A limitation of this study is the finding that although a constant free calcium ion concentration was provided to cells seeded on PLGA/aCaP in excess DMEM and although the expected *ALP* upregulation was confirmed, the typical osteogenic marker gene Runx2 was downregulated. We currently do not know exactly why this was the case. We can only speculate that Runx2 upregulation after a two-week incubation might have taken place but was already over or that the experimental system (excess DMEM) evoked other not anticipated effects, such as a faster downregulation after having triggered upregulation first. In other words, the downregulation of Runx2 after a first quick upregulation might have been accelerated by the special culture conditions used here.

Nevertheless, when we look back at our initial hypotheses of the study, the outcome shows that indeed aCaP nanoparticles incorporated in PLGA evoke an osteogenic commitment in ASCs cultivated in basal medium but only if a constant free calcium ion concentration is provided. We conclude that all possible reactions, including the interaction/equilibrium of the material with the medium, should be considered when in vitro experiments are performed. Above all, when seeding stem cells on an osteoconductive composite material for bone tissue engineering, the free calcium ion concentration in equilibrium with the solid phase has a non-negligible impact on the fate of commitment, in addition to the direct local interaction of the cells with the solid phase, including impacts of surface topography and substrate stiffness. In addition to the significant increase in *ALP* gene expression, the provision of a constant calcium ion concentration also enhanced *CD34* expression significantly. Although *CD34* does not belong to the set of typical osteogenic marker genes, it is notable that a constant calcium ion concentration also affects other commitments in addition to osteogenic commitment, particularly if stem cells are used that exhibit a donor-specific heterogeneity. Thus, in addition to cell–material interactions, it is important to also investigate the impact of the free calcium ion concentration on the cells seeded on a material. The actual calcium ion concentration might be influenced by the solid substrate, particularly if calcium phosphate phases are incorporated. This aspect has to be taken into consideration when osteoinduction of bone-mimicking scaffold materials are discussed.

## 4. Materials and Methods 

### 4.1. Scaffolds

Clinically approved PLGA (85:15) was obtained from Boehringer Ingelheim. Flame spray pyrolysis was used to prepare the aCaP nanoparticles (Ca/P = 1.5), as described earlier [48], using calcium-2-ethylhexanoic salt (synthesized with calcium hydroxide from Riedel de Haen, Ph. Eur. and ethylhexanoic acid from Sigma-Aldrich, Zurich, Switzerland) and tributyl phosphate (Sigma-Aldrich, 98%, Zurich, Switzerland). The preparation of PLGA/aCaP nanocomposites was based on previous protocols [49]. The aCaP nanoparticles were dispersed in chloroform (Riedel de Haen, Ph. Eur.) with 5 wt% Tween20 (polysorbate20, Fluka, Ph. Eur.) using an ultrasonic probe (Ultrasonic Homogenizer FS-450N, Sonorex, Bandelin, Switzerland). The dispersion (PLGA/aCaP = 60/40 wt%) was supplemented with 8 wt% PLGA in chloroform, and the mixture was stirred overnight at room temperature. The PLGA/aCaP dispersions and the pure PLGA solutions were electrospun (IME EC-CLI, feeding rate: 3 mL/h; relative humidity: 50%, distance tip-collector: 15 cm; voltage applied: 20 kV; tip kept in a chloroform airstream) [49]. The surface of the scaffolds was imaged using scanning electron microscopy (SEM, FEI, Nova NanoSEM 450, Baltec, Balzers, Lichtenstein). Scaffolds seeded with cells were fixed with 2.5% glutaraldeyde (Axonlab, Baden, Switzerland) and osmium tetroxide (Sigma Aldrich, Zurich, Switzerland) and dried using critical point drying (Tousimis, Rockville, Maryland) prior to SEM analysis. In order to assess the primary diameter of the nanoparticles (~ 22 nm), we used transmission electron microscopy (TEM, FEI, Philips CM 12, TSS microscopy, New York, NY, USA). Disks with a diameter of 1 cm and a thickness of 500–600 µm were prepared with electrospun PLGA and PLGA/aCaP [32]. Moreover, films were prepared from solvent-casted films for 2D cell culture studies. The films had a diameter of 3 cm. The crystalline phases of hydroxyapatite formation and aCaP were investigated by means of SEM and X-ray diffraction (XRD). XRD patterns were collected on an X’Pert PRO-MPD and analyzed with X’Pert Highscore Plus software. After incubation in DMEM, the XRD samples were washed with deionized water and dried at room temperature prior to measurement.

### 4.2. Non-Cellular Scaffold Incubation

To determine non-cellular hydroxyapatite deposition leading to changes in calcium concentration in the medium, PLGA ± aCaP discs (3D meshes and 2D films, respectively) were incubated in 2 mL DMEM with 10% of FBS and Antibiotic-Antimycotic 1X (Thermo Fisher Scientific, Basel, Switzerland) for two weeks at 37 °C and 5% CO_2_, changing the medium every 3 or 4 days. A control was included using the medium without discs. Sample size was *n* = 3. The calcium concentration remaining in the solution of the samples was measured by a colorimetric assay kit (Sigma-Aldrich, Zurich, Switzerland) using a microplate reader (Tecan, Spark, Männedorf, Switzerland).

### 4.3. Cell Isolation

Human ASCs were isolated from fat tissue with the informed consent of the patient according to Swiss (Züricher Kantonale Ethik-Kommission KEK-ZH: StV 7-2009) and international ethical guidelines (ClinicalTrials.gov Identifier: NCT01218945), as reported by Buschmann et al. [50]. The extraction procedure was performed following Zuk et al. [6] and approved by the Swiss ethical guidelines (KEK-ZH: StV 7-2009). ASCs were characterized according to established procedures [32,51]. Of the 30 isolated primary ASC lines [50], we selected one. This selection was based on previous findings regarding its differentiation capacity—it differentiated easily towards the endothelial cell (EC) phenotype, towards adipocytes and chondrocytes and moderately to well towards osteoblasts (OBs) [31]. The fat for these primary cells had been received from a 29-year-old woman by abdominal liposuction. This donor was chosen because it seemed to be a bigger challenge to evoke an osteogenic commitment in ASCs, induced by the nanocomposite, than in ASCs that had previously shown an easy osteogenic differentiation in the osteogenic culture medium, as tested during multilineage differentiation [33].

### 4.4. Multilineage Cell Differentiation

Lineage-specific differentiation of ASCs towards the osteoblast, the endothelial, the adipogenic and the chondrogenic cell lineage had been achieved previously [33] using cell culture media supplementation according to Zuk et al. [6]. We used Von Kossa and Alizarin red staining in order to semi-quantitatively assess the osteogenic differentiation extent; CD31 immunohistochemical staining to evaluate the endothelial cell differentiation; Alcian Blue staining to determine the ability of the ASCs to differentiate towards chondrocytes; Oil Red O staining to obtain information about the adipogenic differentiation. These four differentiations had been carried out before for the primary cells under view, using induction media, and results were presented earlier [33].

### 4.5. Tissue Engineered Constructs

Before seeding the cells, the PLGA and PLGA/aCaP discs, respectively, were immersed in 5 mL DMEM medium (PAN Biotech, Aidenbach, Germany) containing 10% FBS and 50 µg mL^−1^ gentamycine for 15 min. Then, they were dried in the laminar flow bench. The 1.0 x 10^6^ ASCs were seeded on both sides of the disk. During seeding, the cells were distributed homogenously over the disk surfaces (macroscopic evaluation). All seeded scaffolds were cultivated in 6-well plates using 2 mL DMEM medium with 10% FBS and 50 µg mL^−1^ gentamycine or osteogenic medium (DMEM with 10% FBS, 50 µg ml^−1^ gentamycine, 10 mM beta-glycerophosphate, 50 µM ascorbic-2-phosphate and 100 nM dexamethasone) for 2 weeks in a humidified atmosphere of 95% air and 5% CO_2_ at 37 °C. In addition, 3D PLGA/aCaP meshes were incubated in excess DMEM (20 mL) in a Petri dish with a diameter of 10 cm. The medium was changed every 3 or 4 days. At the end of the experiment (2 weeks), the samples were carefully collected and ¼ was fixed overnight by 4% formalin in phosphate-buffered saline (Kantonsapotheke Zurich), followed by histology. Another ¼ was used for micro-CT analysis and the rest for real-time polymerase chain reaction (PCR). For the film experiments, films were seeded with 1.0 × 10^6^ ASCs and placed on the bottom of a 6-well plate either with osteogenic supplements (OS) or without (DMEM). After 2 weeks of cultivation, cells were collected for PCR. Sample size was *n* = 3.

### 4.6. Gene Expression

Total RNA was extracted from the electrospun meshes or from the films with a RNeasy Mini Kit (Qiagen). The kit was used according to the manufacturer’s instruction. A Nanodrop ND-1000 Spectrophotometer (Witec, Sursee, Switzerland) was used to quantify the RNA. A total of 500 ng RNA was reversed transcribed into cDNA with oligo-dT primers (Invitrogen, Carlsbad, CA, USA), DTT (Invitrogen), dNTP mix (Invitrogen), 5x FSB (Invitrogen), SuperscriptIII (Invitrogen) and RNA inhibitor (Applied Biosystem, Buchs, Switzerland). SYBR^®^ Green (Qiagen, Hilden, Germany) and primers synthesized by Microsynth (Balgach, Switzerland) were used for quantitative PCR. For primer sequences, see SI Table S1. Primers for *CD73*, *CD90* and *CD105* (minimal criteria [52]), for *CD31* and *CD34* (markers of endothelial cells), for *ALP* and *RUNX2* (early osteogenesis), for collagen I (medium osteogenesis) and osteocalcin (late osteogenesis), for *PPAR-γ-2* (adipogenesis) and *Sox9* (chondrogenesis) [53] were used.

### 4.7. Micro-CT

After fixation in 40% ethanol, the wet 3D electrospun scaffolds were scanned in a micro-CT 40 (Scanco Medical, Cressier, Switzerland) operated at 45 kV and 114 μA. Scans were executed using a high-resolution mode, resulting in a voxel size of 8 μm. In reconstructed images, bone tissue was segmented from background using a global threshold of 12% of maximum gray value. The following parameters were determined: the total volume (TV), the bone volume (BV) and the bone volume fraction (BV/TV).

### 4.8. Statistics

The data were analyzed with StatView 5.0.1 software. One-way statistical analysis of variance (ANOVA) was conducted to test the significance of differences between different materials, different culture media and between 2D films and 3D electrospun meshes. Unpaired t-tests were performed to compare ΔCt values of PLGA and PLGA/aCaP systems, respectively. Pairwise comparison probabilities (*p*) were calculated using Fisher’s PLSD post-hoc test to evaluate differences between the groups. *p* values < 0.05 were considered significant (denoted by *); **, *p* < 0.01; ***, *p* < 0.001. Values were expressed as means ± standard deviations.

## Supplementary Materials

Supplementary materials can be found at https://www.mdpi.com/1422-0067/21/7/2627/s1.

## Abbreviations

aCaP	Amorphous calcium phosphate
ASCs	Adipose-derived stem cells
DMEM	Dulbecco’s modified essential medium
OS	Osteogenic induction medium
PLGA	Poly(lactic-co-glycolic acid)
Ca^2+^	Calcium ions
