# Supplementary material for: Directing Stem Cell Commitment by Amorphous Calcium Phosphate Nanoparticles Incorporated in PLGA: Relevance of the Free Calcium Ion Concentration
Source: Int J Mol Sci. 2020 Apr 9;21(7):2627. doi: 10.3390/ijms21072627 (PMC7177725; doi:10.3390/ijms21072627)
Supplement: Supplementary file 1 [file ijms-21-02627-s001.pdf]

Supporting Information

Directing stem cell commitment by amorphous calcium phosphate nanoparticles incorporated in PLGA: Relevance of the free calcium ion concentration

Olivier Gröninger[1], Samuel Hess[2], Dirk Mohn[1,2], Elia Schneider[1], Wendelin Stark[1], Sonja Märsmann[3,4], Petra Wolint[3], Maurizio Calcagni[3], Paolo Cinelli[4] and Johanna Buschmann[3,*]

[1]Institute for Chemical and Bioengineering, Department of Chemistry and Applied Biosciences, ETH Zurich, Zurich, 8093, Switzerland
[2]Clinic for Preventive Dentistry, Periodontology and Cariology, University of Zurich, Center of Dental Medicine, Zurich, 8032, Switzerland
[3]Division of Plastic and Hand Surgery, University Hospital Zurich, Zurich, 8091, Switzerland
[4]Division of Trauma Surgery, University Hospital Zurich, Zurich, 8091, Switzerland

[*]Corresponding author: johanna.buschmann@usz.ch

Word count for abstract: 169

Word count for manuscript: 4117

Number of references: 50

Number of figures: 7

Number of tables: 0

Number of Supplementary online-only files, if any: 1

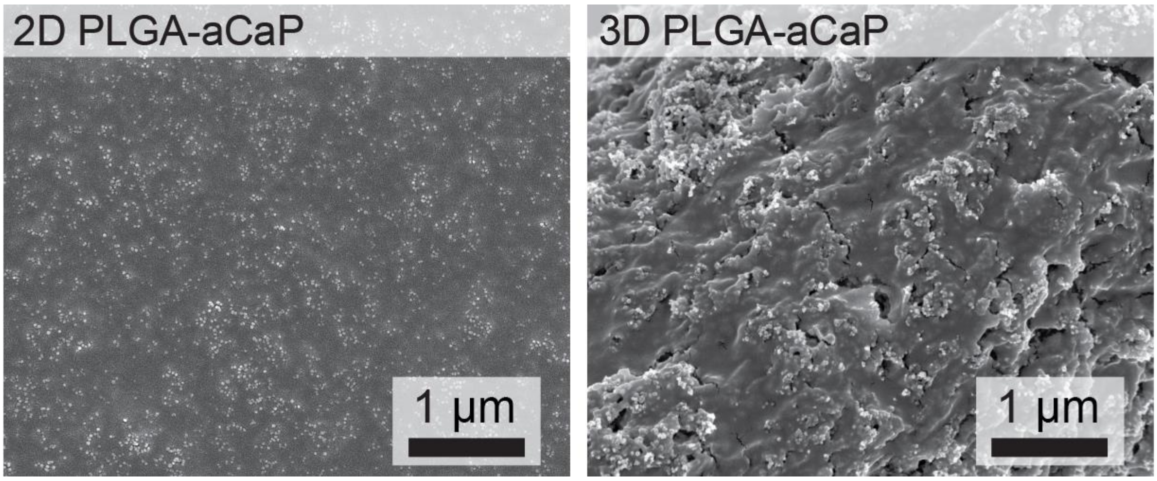

Figure S1. Increased magnification using SEM of 2D and 3D scaffolds to visualize aCaP nanoparticles. The surfaces of the scaffolds are shown.

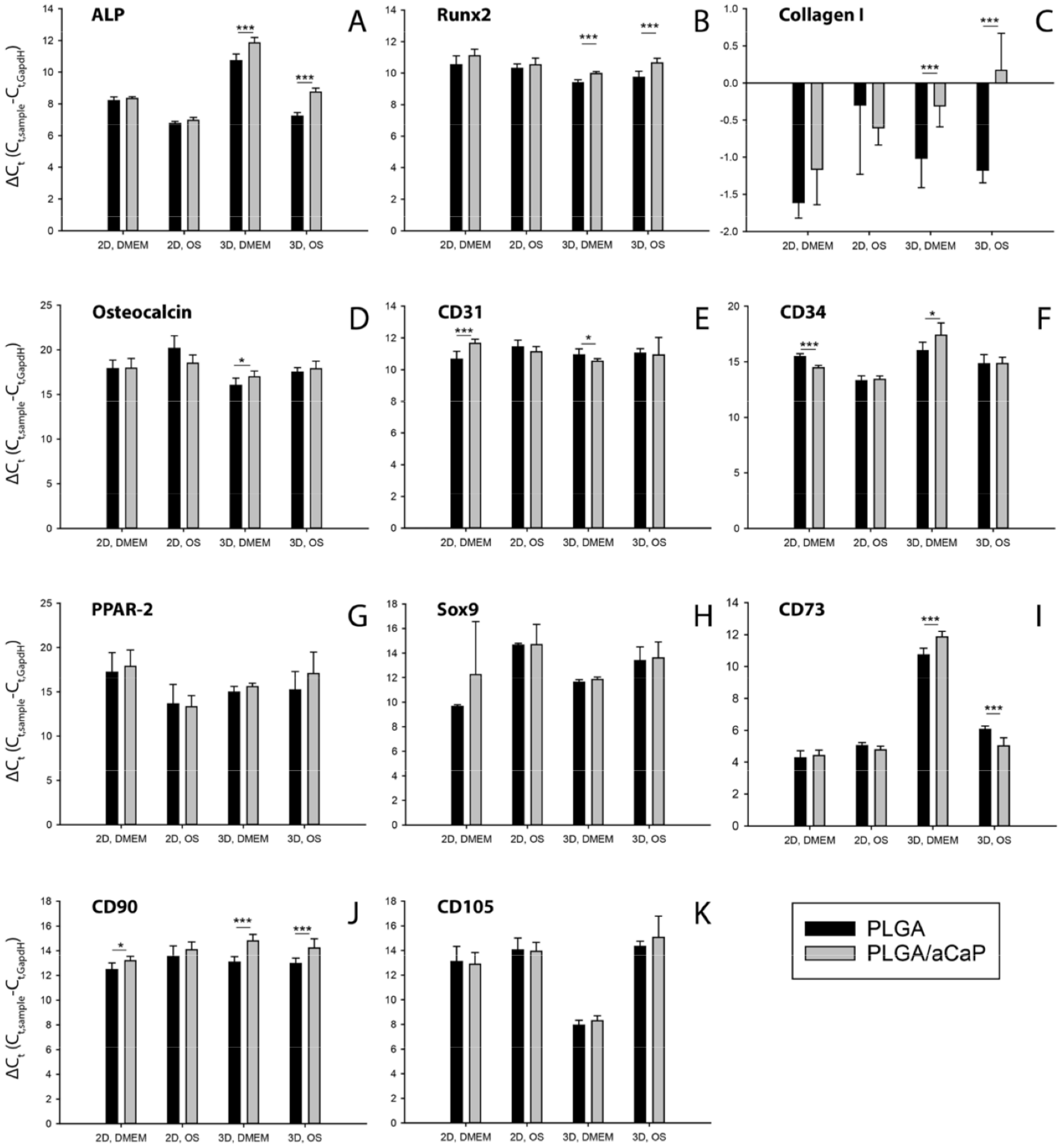

Figure S2. Gene expression of 11 genes (A-K) after a two-week incubation in either DMEM (basal culture medium) or OS (osteogenic medium) for casted films (2D) and electrospun random fiber meshes (3D). Delta Ct values indicate a downregulation if they are increased relatively to each other and vice versa. T-tests were performed to compare delta Ct values of PLGA and PLGA/aCaP for each condition. For comparison of the manifold inductions (PLGA/aCaP vs PLGA, expressed as $2^{-\Delta\Delta Ct}$), we refer to **Figure 3** in the main manuscript.

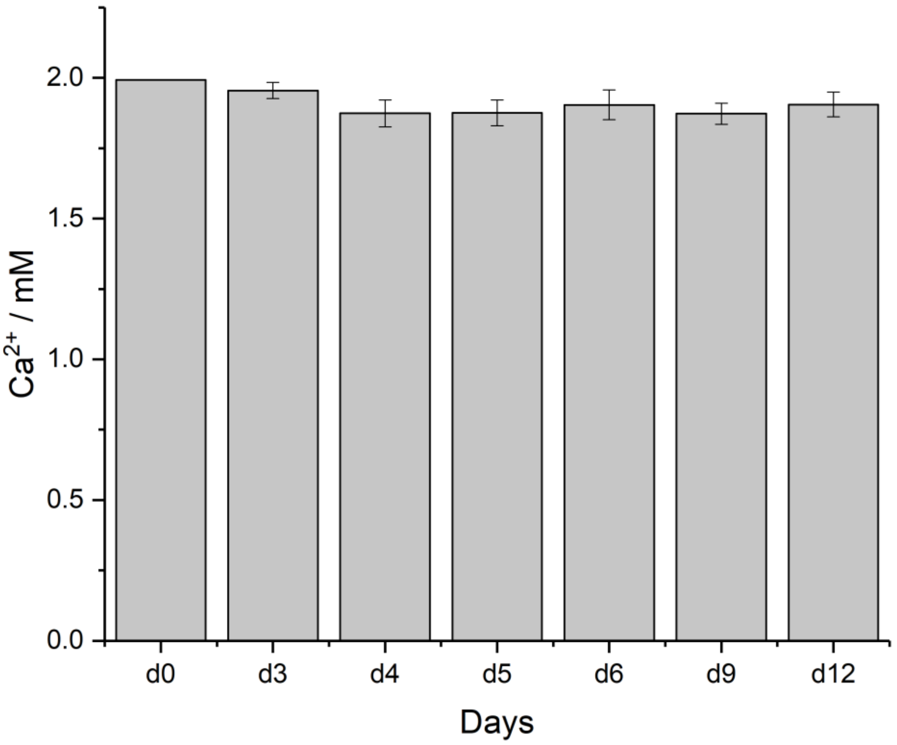

Figure S3. No significant depletion of calcium ions measured using excess (20 mL) DMEM.

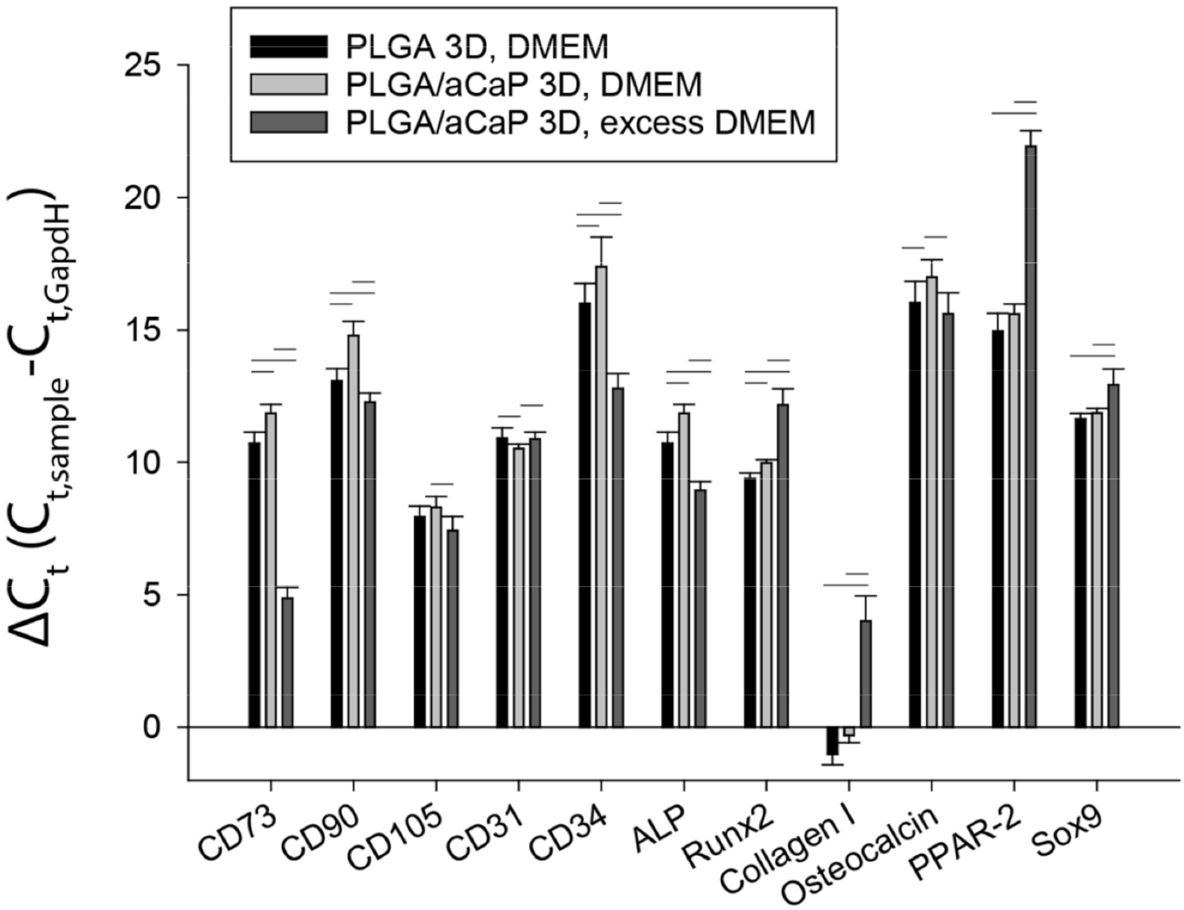

Figure S4. Gene expression for 3D electrospun meshes seeded with human ASCs under different culture conditions. Excess DMEM (10 times more than normal) was used to provide a constant Ca2+ concentration. The lines indicate statistical significance with $p < 0.05$. For comparison of the manifold inductions (PLGA/aCaP vs PLGA, expressed as $2^{-\Delta\Delta Ct}$), we refer to **Figure 3** in the main manuscript.

Gene		Sequence (5'-3')
GAPDH	forward	ACC ACA GTC CAT GCC ATC AC
	reverse	TCC ACC ACC CTG TTG CTG TA
CD31	forward	ATT GCA GTG GTT ATC ATC GGA GTG
	reverse	CTC GTT GTT GG AGT TCA GAA GTG G
CD34	forward	TGA AGC CTA GCC TGT CAC CT
	reverse	CGC ACA GCT GGA GGT CTT AT
CD73	forward	CTC CTC TCA ATC ATG CCG CT
	reverse	CCC AGG TAA TTG TGC CAT TGT
CD90	forward	TGA ATA CAG ACT GCA CCT CCC
	reverse	CTT GAC GGG TGA GGC TAG GA
CD105	forward	CAG CAG TGT CTT CCT GCA TC
	reverse	AGT TCC ACC TTC ACC GTC AC
ALP	forward	CTG GTA GTT GTT GTG AGC AT
	reverse	CCC AAA GGC TTC TTC TTG
Osteocalcin	forward	CAC TCC TCG CCC TAT TGG C
	reverse	CCC TCC TGC TTG GAC ACA AAG
Runx2	forward	GAA CCC AGA AGG CAC AGA CA
	reverse	GGC TCA GGT AGG AGG GCT
COL1	forward	TGA CGA GAC CAA GAA CTG
	reverse	CCA TCC AAA CCA CTG AAA CC
PPAR-□-2	forward	AGG AGC AGA GCA AAG AGG
	reverse	CCT CGG ATA TGA GAA CCC
Sox 9	forward	CTC TGG AGA CTT CTG AAC GAG AGC
	reverse	GTT CTT CAC CGA CTT CCT CCG

Table S1. The sequences of forward and reverse primers.
